# Decreased Volume of Bone Marrow Adipocytes With Sparse Gelatinous Marrow Transformation in a Patient With Pancytopenia With Anorexia Nervosa: A Case Report

**DOI:** 10.7759/cureus.58390

**Published:** 2024-04-16

**Authors:** Masaya Mashimoto, Fumihiro Higuchi, Sakie Okazaki, Yoshinobu Hoshii, Shin Nakagawa

**Affiliations:** 1 Division of Neuropsychiatry, Department of Neuroscience, Yamaguchi University Graduate School of Medicine, Ube, JPN; 2 Department of Pediatrics, Yamaguchi University Graduate School of Medicine, Ube, JPN; 3 Department of Diagnostic Pathology, Yamaguchi University Hospital, Ube, JPN

**Keywords:** pancytopenia, gelatinous marrow transformation, dry tap, bone marrow adipocyte, anorexia nervosa

## Abstract

Patients with anorexia nervosa (AN) often have complications of hematologic abnormalities and pancytopenia, which can be fatal. In patients with AN, the rates of anemia, leukopenia, and thrombocytopenia have been reported as 16.7-39%, 7.9-39%, and 5-11%, respectively; in patients with severe AN, the rates of anemia, leukopenia, thrombocytopenia, and pancytopenia have been reported as 47-83%, 49.5-79%, 16.8-25%, and 16.4-23%, respectively. Hematologic abnormalities are often associated with morphological myeloid transformations such as hypoplasia, aplasia, and gelatinous marrow transformation (GMT). Hypocellularity, such as hypoplastic or aplastic, often results in a dry tap, whereas GMT does not usually result in this because of the aspiration of gelatinous material. Therefore, bone marrow aspiration in patients with pancytopenia with AN usually does not show a dry tap. The bone marrow adipocyte (BMA) volume increases in patients with AN, except in those with severe malnutrition. Patients with AN experiencing pancytopenia often exhibit GMT associated with atrophy of the originally increased volume of BMAs. Herein, we report the case of a patient with pancytopenia with AN who exhibited a dry tap on bone marrow aspiration. A bone marrow biopsy revealed sparse GMT with decreased BMA volume and areas of hematopoietic cells, adipocytes, and no GMT. A 13-year-old Japanese girl weighing 25.8 kg (BMI: 10.0 kg/m^2^) was admitted to our hospital and received nutritional therapy. The patient presented with pancytopenia and fever, prompting the conduct of bone marrow examinations. Bone marrow aspiration resulted in a dry tap, and the bone marrow biopsy revealed sparse GMT with a decreased volume of BMAs. Additionally, an area devoid of hematopoietic cells, adipocytes, or GMT was observed. Nutritional therapy resulted in weight gain and improved pancytopenia. Upon discharge, the patient weighed 40.0 kg (BMI: 15.5 kg/m^2^) with a normal WBC count, hemoglobin levels, and platelet count. It is significant to study hematological and bone marrow changes because patients with AN often present with hematologic abnormalities. The identification of sparse GMT, which is associated with a decrease in BMA volume and the presence of an area devoid of hematopoietic cells, adipocytes, or GMTs, is a novel finding. The improvement in pancytopenia following nutritional therapy suggests a link between myeloid transformation and malnutrition. Consequently, in patients with pancytopenia associated with AN exhibiting these bone marrow findings, nutritional therapy is necessary.

## Introduction

Patients with anorexia nervosa (AN) often experience complications such as hematologic abnormalities and pancytopenia, which can be fatal. In patients with AN, the rates of anemia, leukopenia, and thrombocytopenia have been reported as 16.7-39%, 7.9-39%, and 5-11%, respectively; in patients with severe AN, anemia, leukopenia, thrombocytopenia, and pancytopenia have been reported as 47-83%, 49.5-79%, 16.8-25%, and 16.4-23%, respectively [1-3]. Hematological abnormalities in patients with AN are often accompanied by morphological changes in the bone marrow [1]. In 2002, Abella et al. classified the bone marrow findings in patients with AN into five levels: normal, hypoplastic marrow, aplastic marrow, partial gelatinous marrow transformation (GMT), and complete GMT [4]. Hypocellularity, such as hypoplastic or aplastic, often results in a dry tap, whereas GMT does not usually result in this because of the aspiration of gelatinous material. Therefore, bone marrow aspiration in patients with pancytopenia with AN usually does not show a dry tap. Paradoxically, patients with AN exhibit an elevated volume of bone marrow adipocytes (BMAs), while the subcutaneous and visceral adipose tissues diminish [5]. Initially, BMAs increase in size and number as the hematopoietic tissue diminishes during the hypoplasia and aplasia stages. Although the volume of BMAs is increased in patients with mild-to-moderate AN, those with severe AN often exhibit GMT associated with adipocyte tissue collapse resulting from the reduction in the previously increased BMA volume [6].

It is significant to study hematological changes and bone marrow changes because patients with AN often present with hematologic abnormalities. Patients with pancytopenia with AN sometimes require a bone marrow examination when they present with a fever of unknown cause; however, few bone marrow findings other than GMT have been reported. Herein, we report a new bone marrow finding, where the patient’s bone marrow biopsy showed a sparse GMT, accompanied by a reduced BMA volume and an area devoid of hematopoietic cells, adipocytes, or GMT. Nutritional therapy increased the patient’s weight and improved pancytopenia. Since pancytopenia can be a major clinical challenge, the finding that such myeloid transformation occurs and improves with nutritional therapy is significant.

## Case presentation

A 13-year-old Japanese girl was admitted to our hospital due to her low body weight. She presented with pancytopenia and fever; therefore, a myeloid examination was performed. A bone marrow biopsy revealed a dry tap, sparse GMT, accompanied by a reduced BMA volume, and an area devoid of hematopoietic cells, adipocytes, or GMT. Pancytopenia resolved as her nutritional status and weight improved with nutritional therapy.

The patient had no remarkable medical history or intellectual development abnormalities. The patient weighed 44 kg three months before admission, which coincided with the weight in the previous physical examination. The patient was an accomplished athlete; however, she reported a fear of being mocked by peers for underperforming in sporting events. The patient had a low BMI due to her athleticism but reported no problems with eating. The patient experienced anxiety about being outperformed by her marathon rivals during training sessions two months before admission. Therefore, she trained more and ate less. As a result, she lost weight.

One month before admission, the patient sought medical attention at our hospital due to complaints of weight loss and malaise. The height, weight, BMI, and %mBMI at the time of visit were 160.5 cm, 35.0 kg, 13.6 kg/m^2^, and 70.5% (mBMI of a Japanese girl aged 13 years and 10 months: 19.3 kg/m^2^) [7], respectively. The patient expressed a fear of gaining weight, leading to reduced food intake and increased activity levels. She had not yet reached menarche. Peripheral blood analysis showed normal results, with a platelet (PLT) count of 19.8 × 10^4^/μL, a WBC count of 6,500 cells/μL (no myeloblasts, promyelocytes, or atypical lymphocytes), and a hemoglobin (Hb) level of 13.9 g/dL. The patient was diagnosed with AN according to the Diagnostic and Statistical Manual of Mental Disorders, Fifth Edition. Inpatient treatment was recommended; however, the patient and her family preferred outpatient treatment. Psychoeducation, nutritional guidance, and prescriptions for a high-calorie diet and vitamin B supplements were provided. She limited her club activities but was overactive at home, rarely sitting down. Nutritional guidance was not followed, and her family could not ensure she ate adequately. We explained that if she did not gain weight, she would have to be admitted to the hospital, but she still could not eat due to the fear of gaining weight.

At the age of 13 years and 11 months, she was admitted to our hospital due to severe debilitation and an inability to stand. Upon admission, the patient weighed 25.8 kg with a BMI of 10.0 kg/m^2^, %mBMI of 51.8%, a blood pressure of 88/53 mmHg, a heart rate of 53 bpm, a body temperature of 36.4°C, and a respiratory rate of 44 breaths/min. Peripheral blood analysis revealed thrombocytopenia with a PLT count of 4.3 × 10^4^ cells/μL, a WBC count of 6,170 cells/μL (no myeloblasts, promyelocytes, or dysplastic lymphocytes), and an Hb level of 16.4 g/dL. She presented with dehydration and liver dysfunction. Blood biochemistry tests revealed the following results: creatinine: 0.77 mg/dL; urea nitrogen: 97 mg/dL; uric acid: 9.2 mg/dL; aspartate aminotransferase: 188 U/L; alanine aminotransferase: 203 U/L; total bilirubin: 2.9 mg/dL; direct bilirubin: 0.5 mg/dL; lactate dehydrogenase: 732 U/L; and albumin: 3.9 g/dL. Vitamin B1 and B12 levels were high due to supplementation. Moreover, the folic acid, iron, zinc, copper, and C-reactive protein levels were 13 ng/mL, 132 μg/dL, 99 μg/dL, 56 μg/dL, and 0.01 mg/dL, respectively, with negative results on antinuclear antibody tests. In addition, the activated partial thromboplastin time was 26.4 seconds, the prothrombin time was 32.3%, the fibrinogen level was 103 mg/dL, and the d-dimer level was 4.4 mg/L. Physical examination revealed purpura on the chest and back, with no other hemorrhages, including nasal, oral, or cerebral hemorrhage. Whole-body computed tomography revealed brain atrophy, but no other abnormal findings were observed.

Following admission, nutritional therapy was initiated at 1,248 kcal/day, both orally and intravenously. Since she was debilitated and unable to sit up and eat on her own, high-calorie supplements were administered through a nasogastric tube. Her caloric intake was gradually increased by approximately 200 kcal/day every two to three days. Due to diminished phosphorus and potassium levels after re-nutrition, oral phosphorus and potassium supplements were administered. Because of purpura and thrombocytopenia, we performed frequent peripheral blood analyses. The PLT count, WBC count, and Hb level decreased after admission (Table 1). The PLT count reached 1.3 × 10^4^/μL on day 3, prompting a PLT transfusion. The lowest recorded WBC count was 1,260 cells/μL (neutrophils: 660 cells/μL, lymphocytes: 470 cells/μL, and monocytes: 130 cells/μL) on day 3. The Hb level dropped to 7.5 g/dL on day 14 (mean corpuscular volume: 96.7 fL; mean corpuscular Hb: 31.3 pg). Given the onset of pancytopenia after weight loss, malnutrition was considered a likely contributing factor.

**Table 1:** Measured variables No hematological abnormalities were found at the initial visit. Following admission, PLT and WBC counts were at their lowest on day 3, and Hb and PLT levels were at their lowest on day 13. Blood counts increased as caloric intake and body weight increased. ① At initial visit; ② At admission; ③ PLT transfusion; ④ At discharge Hb, hemoglobin; PLT, platelet

Day	Normal range	-29	-13	-6	0	1	3	4	5	7	11	13	15	21	25	33	39	53	Unit
WBC count	3,300–8,600	6,500	7,180	7,390	6,170	3,600	1,260	1,500	1,570	1,980	2,250	2,810	2,620	5,510	5,340	6,120	7,500	5,770	cells/μL
Hb	11.6–14.8	13.9	15.2	16.9	16.4	14	13.1	11.1	10.6	10.1	7.7	7.5	8.4	8.6	10.3	10.9	12.5	14.1	g/dL
Reticulocytes	0.5–2.5	N/A	N/A	N/A	N/A	N/A	N/A	0.2	0.2	0.2	1.2	6.5	10.8	12.8	10.2	6.2	5.4	2.8	×10^3^ cell/μL
PLT	15.8–34.8	19.8	12.4	9.9	4.3	3.3	1.3	5.5	5.4	8.5	17.1	23	32	33.7	28.9	21.5	22.9	24.4	×10^4^/μL
Event		①			②		③											④	

On day 3, the patient developed a fever of >38°C but had no symptoms suggestive of a respiratory or urinary tract infection. She had no contact with individuals with influenza or COVID-19. Antigen tests for influenza virus and COVID-19, as well as C-reactive protein tests, all yielded negative results. Blood culture also yielded negative results. No antibiotic was used, and acetaminophen temporarily resolved the fever. Given the uncertain etiology of pancytopenia accompanied by fever, a bone marrow examination was required to eliminate the possibility of leukemia or other hematological pathologies. The need for this examination, acknowledged as both necessary and invasive, was explained to the patient and her family. Informed consent was obtained from all participants. On day 5, several pediatric specialists performed multiple bone marrow aspirations on the left and right superior posterior iliac ridges; however, all attempts yielded dry taps. Therefore, a bone marrow biopsy was performed. Nutritional therapy was continued until the results of the bone marrow biopsy were available. On day 13, the patient wanted to eat, so she initiated a light meal. Thereafter, food intake gradually increased. The patient’s activities of daily living also gradually improved. On day 14, she could stand with the support of a nurse.

**Figure 1:**
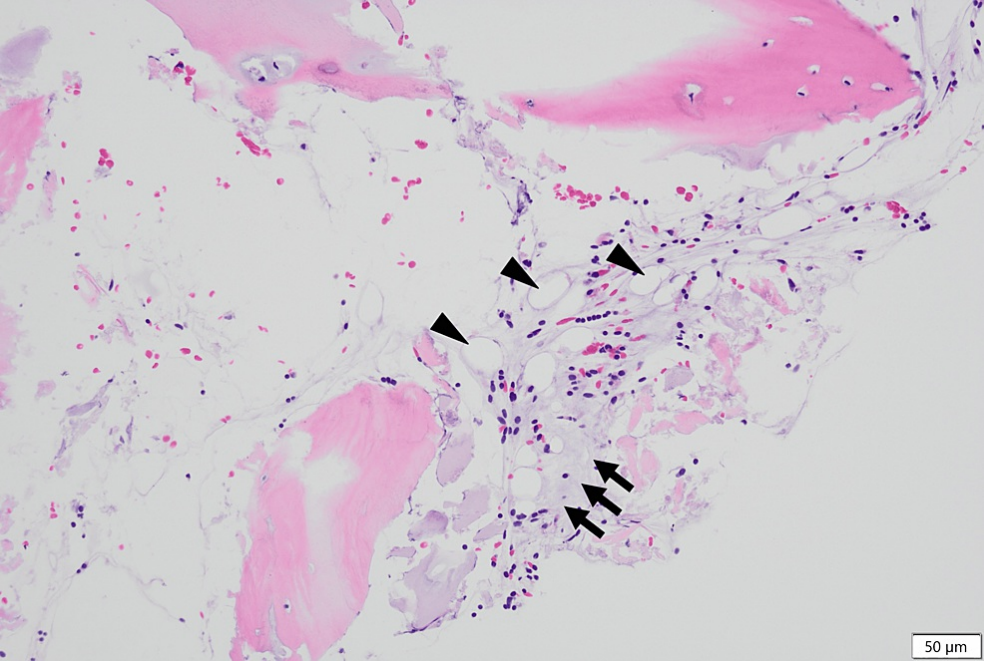
Bone marrow biopsy findings showing partial GMT High-power FOV showing GMT (arrow) present within the intercellular spaces of decreased hematopoietic cells and atrophied adipocytes. Adipocytes (stars) are atrophied and decreased in volume (H&E). FOV, field of view; GMT, gelatinous marrow transformation

**Figure 2:**
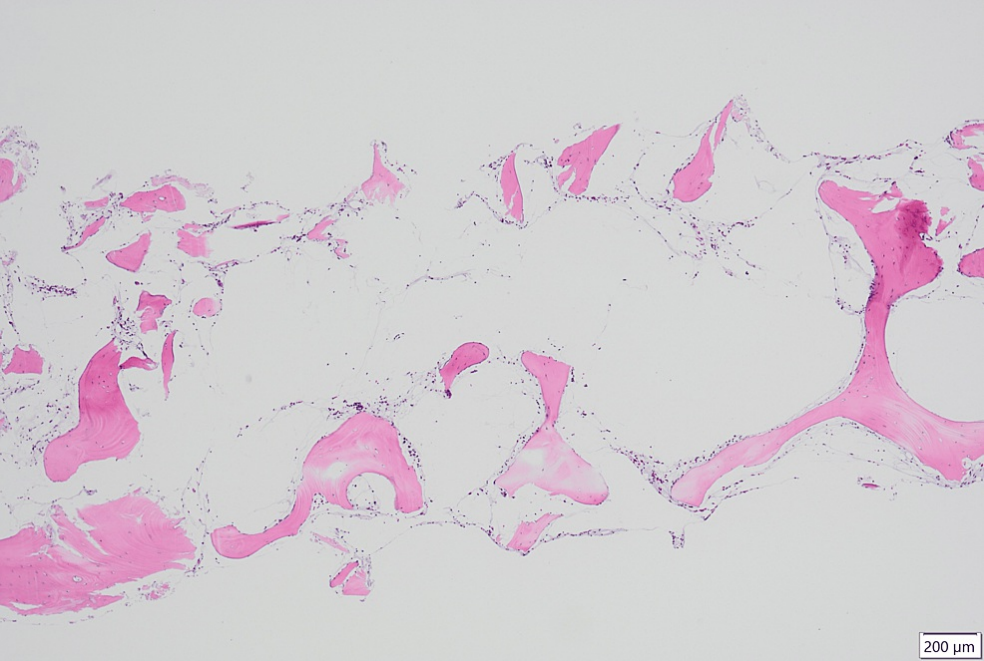
Bone marrow biopsy findings showing an area devoid of hematopoietic cells, adipocytes, or GMT Low-power FOV showing bone marrow with aplasia and no dysplasia. An area devoid of hematopoietic cells, adipocytes, or GMT is observed (H&E). FOV, field of view; GMT, gelatinous marrow transformation

**Figure 3:**
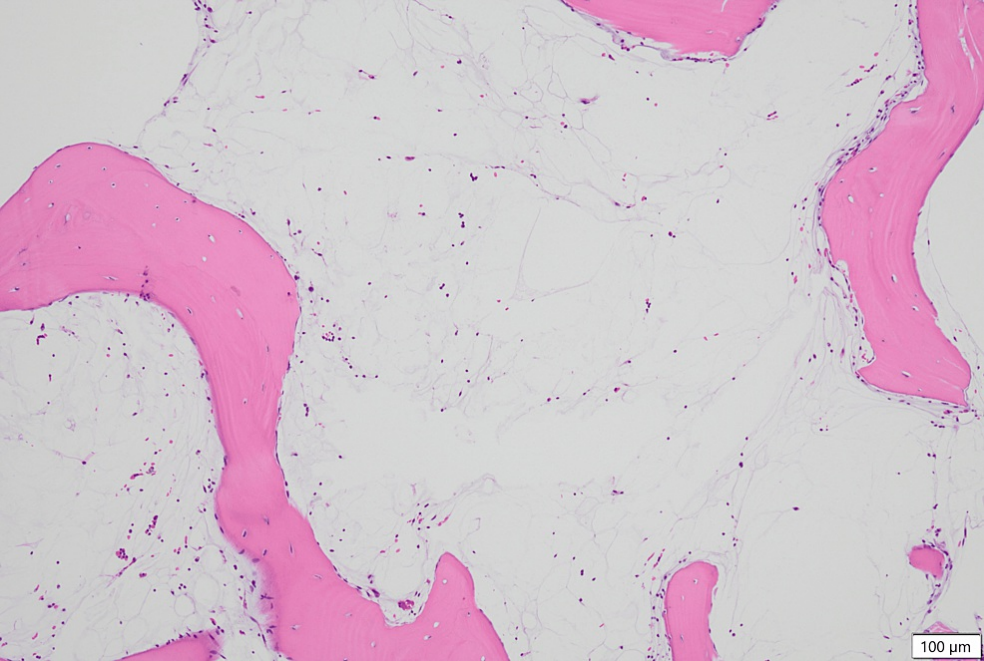
Bone marrow biopsy findings showing adipocytes that have lost their shape Medium-power FOV showing the adipocytes that have lost their shape (H&E). FOV, field of view; GMT, gelatinous marrow transformation

**Figure 4:**
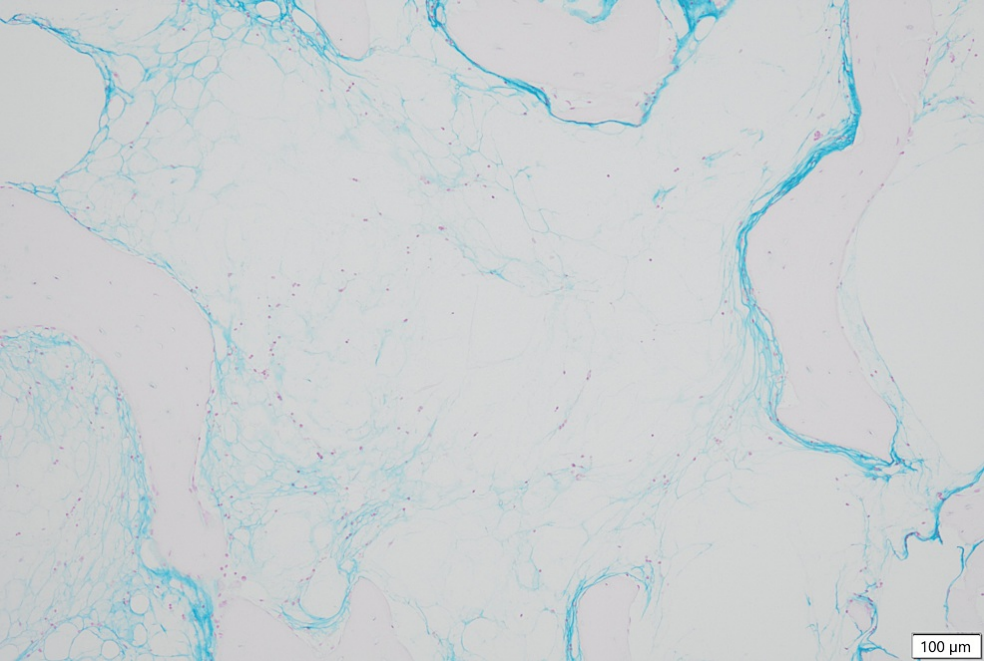
Bone marrow biopsy findings revealing the absence of GMT Medium-power FOV showing that the alcian blue stain is negative and no GMT is present in the area. FOV, field of view; GMT, gelatinous marrow transformation

The results of the bone marrow biopsy, released on day 16, showed aplasia with no dysplasia (Figures 1-4). GMT was observed within the intercellular spaces of hematopoietic cells and atrophied adipocytes (Figure 1). Additionally, an area devoid of hematopoietic cells, adipocytes, or GMT was observed (Figure 2). Notably, the adipocytes appeared to have lost their typical shapes (Figure 3). Alcian blue staining produced a very light stain, although it differed from the staining observed in GMT (Figure 4).

**Figure 5:**
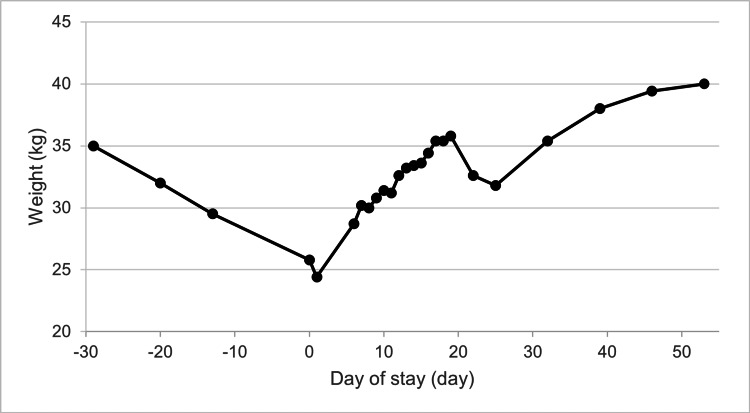
Patient’s weight trajectory over the course of the hospitalization period The timeline is referenced with the date of admission marked as 0 days, the first outpatient visit as −29 days, and the date of discharge as 55 days. The patient’s weight was recorded at 35.0 kg at the initial visit, which decreased to 25.8 kg upon admission and increased to 40.0 kg at the time of discharge. On day 5, bone marrow aspiration and bone marrow biopsy were performed. On day 16, bone marrow biopsy results were available. On day 20, the patient’s weight temporarily decreased due to the improvement of hypervolemia.

On day 16, when the results of the bone marrow biopsy were released, the patient’s weight had reached 33.6 kg and her body temperature was <38°C. Peripheral blood analysis revealed a PLT count of 32.0 × 10^4^ /µL, a WBC count of 2,620 cells/µL, and an Hb level of 8.4 g/dL. Leukemia was ruled out based on the bone marrow findings, and elevated blood counts were observed during this period; therefore, nutritional therapy was continued without the need for additional treatment. The patient had increased his caloric intake to 4,100 kcal in the hope of being discharged earlier. On day 20, the patient lost weight temporarily due to an improvement in hypervolemia but had increased weight throughout admission (Figure 5). Upon discharge on day 55, the patient’s weight had increased to 40.0 kg, with a BMI of 15.5 kg/m^2^ and a %mBMI of 78.6%. At that time, peripheral blood analysis revealed normal levels, with a PLT count of 24.4 × 10^4^ cells/μL, a WBC count of 5,770 cells/μL, and an Hb level of 14.1 g/dL.

We suggested a bone marrow biopsy to confirm bone marrow improvement; however, the patient and family did not consent to a second bone marrow biopsy, citing a perceived lack of clinical benefit. Therefore, we followed up with blood tests. After discharge, the patient’s anxiety was reduced due to no longer being in contact with classmates. As a result, she ate better and gained weight after discharge. At six months after discharge, the PLT count, WBC count, and Hb level were normal. This report complied with the Case Reporting Guidelines [8].

## Discussion

Hematologic abnormalities in patients with AN are often associated with bone marrow changes, and findings on typical bone marrow changes and specific findings such as those in this report are clinically useful. In this case, we observed several noteworthy findings regarding the bone marrow in a patient with pancytopenia and AN. First, the bone marrow biopsy revealed sparse GMT, accompanied by a decrease in BMA volume. Moreover, an area devoid of hematopoietic cells, adipocytes, or GMT was identified. Previous reports have shown increased BMA atrophy with the appearance of GMT [4], but the finding of decreased adipocyte volume in areas without GMT is novel. Second, bone marrow aspiration resulted in a dry tap, which is typically associated with blood disorders such as cancer; however, it is uncommon in patients with AN. There are few reported cases of dry taps in bone marrow aspirations in patients with AN. Finally, the improvement of pancytopenia following nutritional therapy suggested an association between myeloid transformation and malnutrition. Thus, if these bone marrow findings are identified in a patient with pancytopenia with AN, nutritional therapy is necessary.

Myeloid transformation, referred to as GMT, has long been associated with pancytopenia in patients with AN. GMT, also recognized as “starvation marrow” or “serous fat atrophy,” is characterized by focal hypoplasia of adipocytes and hematopoietic cells, accompanied by the accumulation of extracellular gelatinous material primarily composed of hyaluronic acid-based mucopolysaccharides [9]. GMT is not specific to AN and is associated with acute febrile illness, AIDS, alcoholism, malabsorption, cancer, and lymphoma [10]. However, improved nutritional status can reverse GMT in patients with AN [11,12].

In patients with AN, BMAs typically increase, except in those with severe malnutrition [6]. In severe cases, BMAs may undergo atrophy accompanied by the loss of hematopoietic cells in areas affected by GMT [13,14]. Although some studies have reported a decrease in BMA volume associated with GMT [13,15], only a few have documented a decreased BMA volume in areas without GMT. In this case, despite the sparse presence of GMT, a decrease in BMA volume was observed. The decrease in adipocyte volume in the area without GMT differs from that of previous reports.

Additionally, we identified an area devoid of hematopoietic cells, adipocytes, or GMT. Fukudo et al. reported a case of aplastic bone marrow without GMT and the absence of adipocytes [16]. However, they only performed bone marrow aspiration and not a bone marrow biopsy, rendering their bone marrow evaluation inadequate. Takeshima et al. reported bone marrow findings similar to those observed in aplastic anemia patients [17], characterized by an increase in BMA volume, unlike GMT. Therefore, these bone marrow findings differ from those observed in the present patient. We performed a variety of stainings to characterize the contents of this area. However, the results of periodic acid-Schiff and immunohistochemical staining using the S100 protein antibody were negative. Alcian blue staining produced a very light stain, although it differed from the staining observed in GMT (Figure 4). Since the immunohistochemical staining with the S100 protein antibody revealed negative results, we hypothesized that adipocytes were absent, replaced by an extracellular matrix.

The failure to aspirate on bone marrow aspiration is termed “dry tap.” Dry tap may occur owing to marrow fibrosis, markedly hypercellular marrow, hypocellularity with increased adipose tissue, neoplastic infiltration, or primary bone disorders. Myeloid transformation in patients with AN is typically hypoplastic or aplastic and GMT. Hypocellularity such as hypoplasia and aplasia often reveals dry tap. GMT is a more severe myeloid transformation but does not reveal a dry tap because a gelatinous substance is usually aspirated [4]. Bone marrow aspiration in patients with AN does not usually indicate a dry tap, but in the present case, it did. Because the patient was 13 years old, myelofibrosis was unlikely, and blood diseases such as aplastic anemia and leukemia had to be ruled out. Donald and Kakkar reported that among 2,768 patients who underwent bone marrow aspiration, 223 (8.0%) experienced dry taps. Of these patients, 164 (73.5%) had hematological malignancies, 33 (14.9%) had benign hematologic diseases, and 9 (4%) had non-hematologic diseases [18]. Furthermore, among hematological malignancies, leukemia was the most common cause of dry taps, occurring in 40.3% of patients [18]. To our knowledge, Saito et al. conducted the only study that reported dry tap in patients with pancytopenia with AN [19]. In their patient, a bone marrow biopsy revealed decreased hematopoietic function and megakaryocytes; however, they did not evaluate adipocytes or GMT. In the present patient, we speculated that bone marrow aspiration revealed a dry tap due to failure of aspiration with few hematopoietic cells and GMT. It is important to note that bone marrow aspiration in a patient with pancytopenia with AN indicates a dry tap.

In the present patient, pancytopenia persisted despite the initiation of re-nutrition. Funayama et al. reported that the PLT count, WBC count, and Hb level decreased after admission and were the lowest on days 5-10 [2]. Their findings aligned with those observed in our patient, where hematological abnormalities peaked after the initiation of nutritional therapy.

However, this case report had certain limitations. First, a second bone marrow biopsy was not performed. Although we observed an improvement in pancytopenia, we could not confirm whether the bone marrow findings had also improved. We anticipate that the impact of nutritional therapy on BMAs will become evident in the future. Second, we are still exploring the composition of an area devoid of hematopoietic cells, adipocytes, or GMT. The occurrence of a dry tap during bone marrow aspiration suggests the presence of a substance that could not be aspirated. Although various stains provided some clues, we were ultimately unable to identify the exact composition of this substance. We hope to elucidate the substances present in areas devoid of hematopoietic cells, adipocytes, and GMT, as well as the causes of these myeloid transformations, such as the duration of disease, degree of underweight, and nutrient deficiencies.

## Conclusions

We report a rare bone marrow finding in a patient with pancytopenia associated with AN. Bone marrow aspiration resulted in a dry tap, while bone marrow biopsy revealed sparse GMT, accompanied by a reduced BMA volume and an area devoid of hematopoietic cells, adipocytes, or GMT. Nutritional therapy improved pancytopenia in the context of myeloid transformation. Identifying such bone marrow findings underscores the importance of nutritional therapy in managing pancytopenia in AN. Long-term follow-up is needed to assess the sustainability of the observed improvement and potential relapses. Good nutritional status must be maintained to sustain improvement in the patient’s hematologic status.
